# Insects with similar social complexity show convergent patterns of adaptive molecular evolution

**DOI:** 10.1038/s41598-018-28489-5

**Published:** 2018-07-10

**Authors:** Kathleen A. Dogantzis, Brock A. Harpur, André Rodrigues, Laura Beani, Amy L. Toth, Amro Zayed

**Affiliations:** 1 Department of Biology, York University, 4700 Keele St., Toronto, Ontario Canada; 2 Donnelly Centre, University of Toronto, Toronto, Canada; 3 Departamento de Biologia, Faculdade de Filosofia Ciências e Letras de Ribeirão Preto, Universidade de São Paulo, São Paulo, Brazil; 4 Dipartimento di Biologia, Università di Firenze, Florence, Italy; 5 Department of Ecology, Evolution & Organismal Biology and Entomology, Iowa State University, Ames, USA

## Abstract

Eusociality has independently evolved multiple times in the hymenoptera, but the patterns of adaptive molecular evolution underlying the evolution and elaboration of eusociality remain uncertain. Here, we performed a population genomics study of primitively eusocial *Polistes* (paper wasps), and compared their patterns of molecular evolution to two social bees; *Bombus* (bumblebees), and *Apis* (honey bees). This species triad allowed us to study molecular evolution across a gradient of social complexity (*Polistes* < *Bombus* < *Apis*) and compare species pairs that have similar (i.e. *Polistes* and *Bombus*) or different (i.e. *Polistes* and *Apis*) life histories, while controlling for phylogenetic distance. We found that regulatory genes have high levels of positive selection in *Polistes*; consistent with the prediction that adaptive changes in gene regulation are important during early stages of social evolution. *Polistes* and *Bombus* exhibit greater similarity in patterns of adaptive evolution including greater overlap of genes experiencing positive selection, and greater positive selection on queen-biased genes. Our findings suggest that either adaptive evolution of a few key genes underlie the evolution of simpler forms of eusociality, or that the initial stages of social evolution lead to selection on a few key traits orchestrated by orthologous genes and networks.

## Introduction

Understanding the origin and elaboration of eusociality is a major goal of evolutionary biology. Eusociality has independently evolved several times in the Hymenoptera^1^ and is characterized by overlapping generations, cooperative brood care, and reproductive division of labour^2–4^. The expression of eusociality is highly variable and ranges from subsocial species characterized by small nests and no caste system but well developed parental care, to highly advanced eusocial species that are characterized by large colonies and highly specialised castes^3,5^. Eusocial species can thus be categorized along a gradient of social complexity typified by traits that include colony size, extent of caste divergence, behavioural specialization, and mode of colony reproduction. However, this gradient does not necessary indicate a tendency for social lineages to become increasingly complex over evolutionary time.

There has been growing interest in identifying the mechanisms and patterns of molecular evolution associated with the rise and elaboration of eusociality. Some researchers have postulated that changes in gene regulation, particularly regulating phenotypic plasticity, are essential for eusocial evolution since it allows individuals to maximize their fitness by adapting to fluctuating environmental and social conditions^6–12^. Other researchers have raised the importance of protein coding evolution, especially of novel genes that underlie novel phenotypic traits found in some social species^13–16^. More recently, it was postulated that different patterns of molecular evolution can be involved during the different stages of social evolution; termed the “social ladder hypothesis”^17,18^. Earlier stages of sociality are predicted to involve prominent changes in gene regulation, while intermediate stages are predicted to involve fixed genomic changes in regulatory sequences and protein coding genes. Finally, later stages of sociality are expected to show greater genome and adaptive changes including the appearance and diversification of novel genes that control specialized social traits^17,18^.

These above hypotheses have been integral for formulating the framework for eusocial evolution, but until recently, were challenging to address since it was difficult to estimate patterns of positive selection across the genomes of multiple non-model organisms. With diminishing sequencing costs and advancements in bioinformatic methods, it is possible now to sequence many individual genomes per species and use this data to estimate patterns of adaptive evolution^19,20^. Previous phylogenetic comparisons of several social and solitary bees and ants have provided a wealth of knowledge on the types of genes that show accelerated patterns of protein sequence evolution in eusocial insects^14,21^. However, it is difficult to directly quantify the selection coefficient on amino-acid changing mutations from such studies^20,22,23^. Population genomic studies provide robust methods for directly quantifying patterns of selection on genes and regulatory sequences^12,16,24,25^. To date, such studies have focused on either highly eusocial species^16,25^ or multiple species with non-independent origins of sociality^24^.

**Figure 1:**
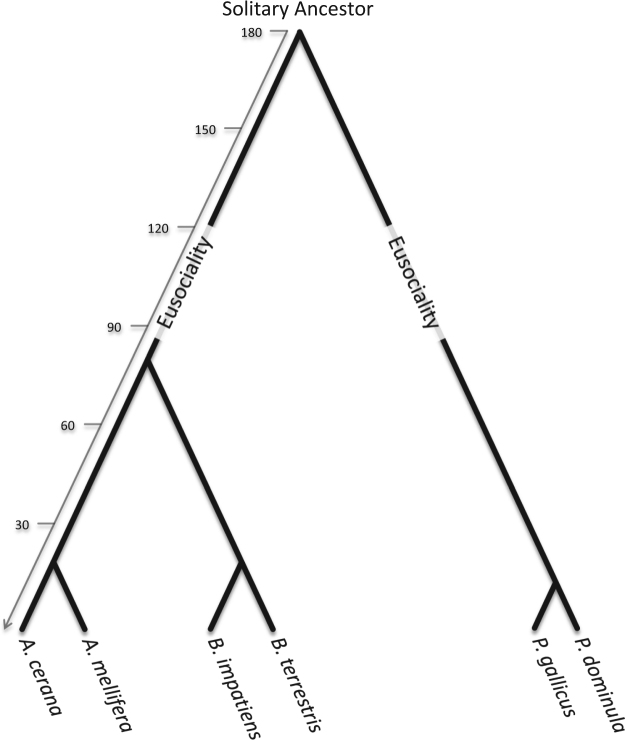
Evolutionary relationships between *Polistes*, *Apis*, and *Bombus*. The divergence of *Apis* and *Bombus* is approximately 80–50 MYA while Apidae diverged from Vespidae ~150–180 MYA, suggesting a total divergence of 300–360 MY. The figure highlights two independent origins of eusociality; the first before the divergence of *Apis* and *Bombus* within the Apinae lineage and the second occurred in the Vespidae lineages, after the divergence from Apidae. Divergence time estimates in MYA. Diagram adapted from estimates in^1,39,58,59^.

Here, we present a comparative population genomic study involving *Apis* (honey bees), *Bombus* (bumblebees), and *Polistes* (paper wasps). *Polistes* of the family Vespidae display an intermediate level of social behaviour referred to as primitive eusociality. Both *Polistes* and *Bombus* are considered to be less socially complex relative to *Apis*, and share similar life history traits including; a solitary founding phase, small colony sizes, gyne (pre-queen) overwintering, establishment of dominance hierarchies, and less distinct caste divergence^3,5,26–28^ (Table 1). Despite the similarities between *Polistes* and *Bombus*, *Bombus* is more socially complex. For example, *Bombus* workers can only lay haploid eggs^29^, while *Polistes* workers retain the potential to mate and lay fertilized diploid eggs^26^. In addition, *Bombus* possess morphologically distinct queen and worker castes, whereas *Polistes* queens and workers show no morphological differences and females retain caste totipotency into adulthood^30^. A*pis*, comparatively, is a highly advanced eusocial bee with large colony sizes, swarm founding, and distinct reproductive division of labour^3^ (Table 1). Like *Bombus*, *Apis* workers can activate their ovaries in the absence of a queen to produce haploid drone eggs. *Apis* and *Bombus* are both corbiculate bees of the subfamily Apinae where eusociality has evolved once^1,31,32^. Vespidae is thought to have diverged from the family Apidae 180–150 million years ago, which has resulted in an equal divergence time between *Polistes* and *Bombus*, and *Polistes* and *Apis*^1^ (Fig. 1). The species triad studied herein allows us to compare patterns of adaptive molecular evolution in two eusocial lineages with either similar (*Polistes* vs. *Bombus*) or different (*Polistes* vs. *Apis*) life histories and social complexity while controlling for divergence time.

**Table 1:** Traits associated with social complexity.

Trait	*Apis*	*Bombus*	*Polistes*
Reproductive Division of Labour	Present	Present	Present
Cooperative Brood Care	Present	Present	Present
Overlapping Generations	Present	Present	Present
Founding	Swarm	Foundress	Foundress
Colony Size	Large	Small	Small
Gynes	Absent	Present	Present
Dominance Hierarchy	Absent	Present	Present
Overwintering	Colony	Solitary	Aggregation
Caste Divergence	High	Intermediate	Low
Totipotent Workers	Absent	Absent	Present

Similarities and differences between colony and life history traits of *Apis*, *Bombus*, and *Polistes*.

We first performed a population genomic analysis of newly sequenced *P*. *dominula* (n = 10), and *P*. *gallicus* (n = 2) diploid workers to estimate patterns of positive selection in paper wasps, and then compared the dataset to published genomic studies on *Apis*^16^ and *Bombus*^24^. The social ladder framework predicts greater similarities in the functions of genes under positive selection and enrichment of shared genes with signs of adaptive evolution between species with similar levels of social complexity (*Polistes* and *Bombus*) compared to those with different levels of social complexity (*Apis*). Qualitatively and quantitatively, we found greater similarities in patterns of adaptive molecular evolution between *Polistes* and *Bombus*. We also addressed the relationship between caste biased gene expression and patterns of adaptive evolution in the species triad. Interestingly, we found evidence that queen-biased genes show more adaptive evolution than worker-biased genes in *Bombus* and *Polistes*, the opposite pattern from previous studies of highly eusocial species.

## Results and Discussion

### Functions of genes experiencing adaptive evolution differ between eusocial species

We used a Bayesian implementation of the McDonald-Kreitman (MK) test to estimate selection across the *Polistes* genome, and compared these levels to published estimates for *Apis* and *Bombus*^16,24^. The MK test quantifies adaptive evolution over the time-scale of species divergence by comparing patterns of synonymous and non-synonymous polymorphisms and divergence between two closely related species to estimate γ, the average selection coefficient on non-synonymous mutations scaled by the effective population size. We calculated γ for 9668 (81.8%) out of 11815 annotated genes in *Polistes dominula* (PdomGDB r1.2)^33^. We found 988 genes (10.45%) with signs of positive selection (γ > 1) (Supplementary Fig. S1). Genes that showed signs of positive selection (γ > 1) in *Polistes* were enriched for functional clusters associated with transcription and gene expression, particularly proteins containing zinc finger domains and RNA polymerases. Additionally, there were clusters of genes associated with fatty acid synthesis and metabolism, post-transcriptional regulation, and metal binding. The most significant term following false discovery rate correction was RNA 3′-end processing (GO:0031123).

The gene functions of adaptively evolving loci in *Polistes* are largely distinctive from those previously discovered for *Bombus* and *Apis*. In *Bombus* enriched GO terms are primarily associated with metabolic functions^24^, while *Apis* genes are enriched for terms associated with behaviour and sensory perception^16^. Though functional enrichment patterns of adaptively evolving genes are divergent between species, they are consistent with the social ladder framework. The social ladder framework postulates that for early to intermediate stages of social evolution, changes in patterns of gene regulation should be prominent^17,18^. This includes adaptive changes to genes such as transcription factors, but can also involve changes in non-coding sequences such as cis-regulatory regions, transcription factor binding sites, and microRNAs^12,21,34^. Our study lends support to this inference as many of the genes found to be under positive selection in primitively eusocial *Polistes* have functions associated with the regulation of gene expression. We found a significantly higher proportion of zinc finger proteins with signs of positive selection, which regulate gene expression by binding to DNA and controlling transcription of target genes^35^, in *Polistes* (n = 62), relative to *Bombus* (n = 53) (χ^2^ = 14.60, *p* = 1.33e^−4^) and *Apis* (n = 15) (χ^2^ = 25.13, *p* = 5.37e^−7^), and we found a significantly higher proportion of genes associated with RNA polymerase under positive selection in *Polistes* (n = 54), relative to *Bombus* (n = 49) (χ^2^ = 10.68, *p* = 1.09e^−3^) and *Apis* (n = 23) (χ^2^ = 9.75, *p* = 1.79e^−3^). We also found evidence for positive selection on a greater proportion of genes related to polyadenylation in *Polistes* (n = 11), relative to *Bombus* (n = 4) (χ^2^ = 7.12, *p* = 7.62e^−3^) and *Apis* (n = 2) (χ^2^ = 4.27, *p* = 0.039), and 3′ end processing in *Polistes* (n = 20) relative to *Bombus* (n = 10) (χ^2^ = 10.36, *p* = 1.29e^−3^) and *Apis* (n = 5) (χ^2^ = 6.76, *p* = 9.33e^−3^). Both processes are important for mRNA processing.

Of the three species studied herein, *Polistes* exhibit the most primitively eusocial societies with the most flexible castes; foundresses perform foraging and reproductive tasks during early colony stages, and workers maintain the ability to transition to reproductive queens into adulthood^5,26^. Flexibility in gene regulation contribute to the phenotypic plasticity of the species, allowing individuals to enhance their fitness during critical events during the colony lifetime, such as queen death^36^. Our data lends support to the importance of gene regulatory evolution in this primitively eusocial group of wasps, an idea which was also suggested from *de novo* genome sequencing of another *Polistes* species^37^.

### Less socially complex species share greater overlap of orthologous genes undergoing adaptive evolution

Eusociality is a model example of convergent evolution as it has evolved independently over multiple evolutionary lineages^1,4,38^. It may be that positive selection on a few keys genes gave rise to eusociality in different lineages. Thus far, studies comparing genes under positive selection across multiple eusocial lineages have not found a high degree of overlap between genes experiencing positive selection in different eusocial species^21,24^.

**Figure 2:**
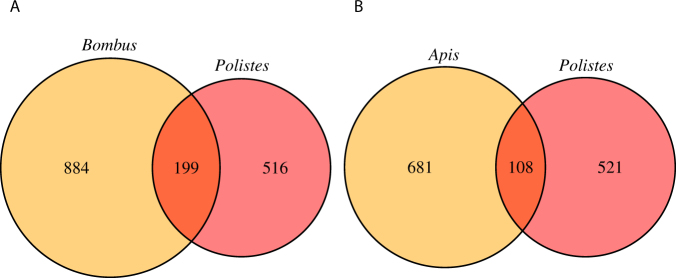
Venn diagram of overlapping genes between *Polistes*, *Bombus*, and *Apis*. (**A**) Overlap of genes with positive selection (γ > 1) between *Bombus* and *Polistes*, (**B**) Overlap of genes with positive selection (γ > 1) between *Apis* and *Polistes*. The proportion of genes that overlap between *Bombus* and *Polistes* is significantly higher relative to the proportion of genes that overlap between *Polistes* and *Apis* (Fisher Exact Test *p* = 2.79e^−10^).

We compared the proportion of overlapping genes under positive selection in *Polistes*, *Apis*, and *Bombus* (Supplementary Dataset 1). We hypothesized that if there is a common set of genes influencing the evolution of eusociality, we expect greater similarity of genes under positive selection between *Polistes* and *Bombus*, relative to *Polistes* and *Apis* due to similarities in life history and colony traits. We found the greatest overlap of genes under positive selection (n = 299) between *Apis* and *Bombus* (Fisher Exact Test (one tail) *p* = 2.2e^−16^). However, this is not surprising given that *Bombus* and *Apis* are closely related, and shared a common ancestor ca. 80 MYA^39^. We found a significant overlap of 199 genes (12.4%) with signs of positive selection between *Polistes* and *Bombus* (Fisher Exact Test (one tail) *p* = 1.01e^−15^) (Fig. 2A), and a significant overlap of 108 genes (8.2%) (Fisher Exact Test (one tail) *p* = 2.28e^−07^) between *Polistes* and *Apis* (Fig. 2B). Overall, there were far more common genes between *Polistes* and *Bombus* relative to *Polistes* and *Apis* (Fisher Exact Test *p* = 2.79e^−10^). Finally, the three-way comparison between *Polistes*, *Bombus*, and *Apis* revealed a higher than expected overlap of genes under positive selection across all three species (n = 31) (χ^2^ = 84.83, *p* =  < 0.0001).

Our results highlight that there is greater overlap of genes undergoing adaptive evolution between *Polistes* and *Bombus* compared to *Polistes* and *Apis*. This is a compelling result given that Vespidae diverged from Apidae 180–150 MYA, and the evolutionary distance between *Polistes* and *Bombus* is similar to that of *Polistes* and *Apis*^1^ (Fig. 1). The finding that species with similar life history traits and social complexity (Table 1) have greater similarity in patterns of adaptive evolution suggests that earlier stages of social evolution may be driven by positive selection on a few common genes, or that being eusocial can drive patterns of adaptive evolution for a few orthologues genes. Both scenarios highlight a much greater association between social complexity and patterns of molecular evolution than previously anticipated.

### Queen traits have greater influence on adaptive evolution in less socially complex species

In addition to studying if similar genes and gene functions experience adaptive evolution in our eusocial species triad, we also quantified the extent of positive selection on queen-biased and worker-biased genes in *Polistes* and compared the patterns to *Bombus*^24^ and *Apis*^16^. Caste-specific phenotypes are likely orchestrated via caste-biased gene expression^9,40^ and thus, our population genetic analysis of caste-biased genes provides insights on the relative contribution of queen and worker traits to fitness and adaptation in different eusocial lineages.

**Figure 3:**
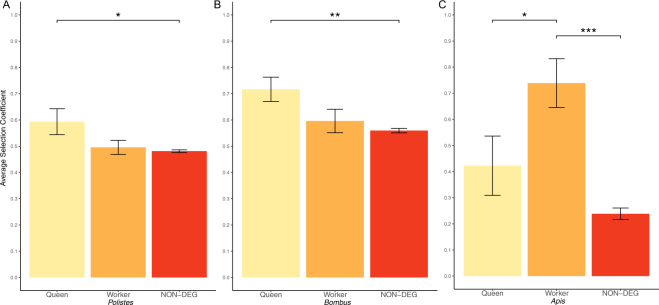
Selection on caste-biased genes in (**A**) *Polistes*, (**B**) *Bombus*, and (**C**) *Apis*. Queen-biased genes have the highest γ and the highest proportion of genes with γ > 1 in both *Polistes* and *Bombus*. *Apis* exhibits an opposite pattern where worker-biased genes have the highest γ and the highest proportion of genes with γ > 1 (see Results for details). The error bars represent the standard error of the mean. NON-DEG = Non-differentially expressed genes. * < 0.05, ** < 0.01, *** < 0.001.

We quantified expression patterns of six *Polistes dominula* queens and five *Polistes dominula* workers^33^ to identify queen-biased (upregulated in queens) and worker-biased (upregulated in workers) genes. Using our population genomic data we were able to estimate the selection coefficient for 7612 out of 8946 genes with transcriptomic data. We observed a trend that queen-biased genes had a higher average γ (0.593 ± SEM 0.0493, n = 114) compared to worker-biased genes (0.496 ± SEM 0.0268, n = 287) and non-differentially expressed genes (0.481 ± SEM 0.00517, n = 7211) (Fig. 3A). Average γ significantly differed among groups (F_2,7609_ = 3.735, *p* = 0.024), with queen-biased genes being significantly higher than non-differentially expressed genes (Tukey *p* = 0.020), but not worker-biased genes (Tukey *p* = 0.113). Worker-biased genes were not significantly different than non-differentially expressed genes (Tukey *p* = 0.858). Furthermore, the proportion of genes with evidence of positive selection (γ > 1) was significantly higher in queen-biased genes (19.3%, n = 22) relative to worker-biased genes (11.1%, n = 32) (χ^2^ = 4.02, df = 1, *p* = 0.045).

The higher incidence of positive selection on queen-biased genes in *Polistes* is similar to patterns observed in *Bombus*^24^. Harpur, *et al*.^24^ found higher levels of average γ, and a high proportion of genes with γ > 1, in genes that are significantly upregulated in reproductively-active (queens and foundresses) females relative to non-reproductively active females (gynes and workers) in bumblebees. To make the *Polistes* and *Bombus* dataset comparable, we reanalyzed the *Bombus* dataset focusing only on queen-biased (n = 185) and worker-biased genes (n = 192)^24,41^ (Fig. 3B). Similar to *Polistes*, we found a trend in which queen-biased genes have a higher average γ (0.717 ± SEM 0.046) relative to worker biased (0.596 ± SEM 0.045) and non-differentially expressed genes (0.559 ± SEM 0.008). Average γ significantly differed among groups (F_2,5640_ = 6.148, *p* = 0.00215), with queen-biased genes significantly higher that than non-differentially expressed genes (Tukey *p* = 0.0017), but not significantly different from worker-biased genes (Tukey *p* = 0.135), and worker-biased genes were not significantly different than non-differentially expressed genes (Tukey *p* = 0.693). Additionally, the proportion of genes with signatures of positive selection (γ > 1) was significantly higher in queen-biased genes (31.4%, n = 58) relative to worker-biased genes (17.7%, n = 34) (χ^2^ = 7.19, df = 1, *p* = 0.007) in *Bombus*, similar to *Polistes* (see above).

The enrichment of queen-biased genes among adaptively evolving genes in *Polistes* and *Bombus* are in stark contrast to *Apis* where worker-biased genes show the greatest levels of positive selection. This includes a greater average selection coefficient (F_2,1688_ = 15.8, *p* = 1.6e^−07^) relative to queens (Tukey *p* = 0.045) and non-differentially expressed genes (Tukey *p* = 2.0e^−07^) (Fig. 3C), and a greater proportion of genes undergoing adaptive evolution (γ > 1) in worker-biased genes relative to queen-biased genes (χ^2^ = 12.81, df = 1, *p* = 0.0003)^16^.

There are two possible explanations for the observed similarities of adaptive evolution in caste-biased genes between *Polistes* and *Bombus*, in contrast to *Apis*. Both *Polistes* and *Bombus* are independently founding; nests are initiated by foundresses, who – if successful in establishing a nest – become the future queen. In contrast, *Apis* is swarm-founding; colonies are established by swarms of bees that include a mated queen and several thousand workers. For independent founding species, there are likely strong selective pressures on future queens to find and establish a nest, forage, and lay eggs^42,43^. There is often very high mortality rates at this stage of colony development^44^. These selection pressures may lead to higher levels of positive selection on genes expressed by queens. Alternatively, the relative contribution between worker-biased and queen-biased genes to adaptive evolution may be influenced by the number of workers within a colony, or the proportion of time that workers are present during a eusocial species’ life cycle^24^.

Recent population genomic work on the advanced eusocial pharaoh ant, *Monomorium pharaoni*s revealed similar patterns to *Bombus* and *Polistes* where queen-biased genes experienced the greatest rates of adaptive evolution^25^. Pharaoh ants, unlike *Bombus* and *Polistes*, found nests by budding (colony fission, Schmidt *et al*.^45^). However, like *Bombus* and *Polistes*, they have smaller nests of approximately 1200 individuals^45^; significantly smaller than *Apis* colonies that typically contain 40,000 to 60,000 workers^46^. Additionally, pharaoh ant colonies are polygynous, maintaining multiple queens per colony, and queen production increases when colony size is reduced^45^. The emphasis on queen numbers and their input to colony growth may influence the patterns of adaptive evolution seen in pharaoh ants. More comparative work using a greater number of species with varying social complexity is needed to better understand how colony founding, colony size, and other life history and eusocial traits influence patterns of adaptive evolution of caste-biased genes. This will enable us to fully understand the molecular processes involved in the evolution and elaboration of eusociality, and study the relationship between eusocial evolution and molecular evolution.

## Conclusion

Our comparative analyses have demonstrated that there are convergent patterns of adaptive molecular evolution associated with different levels of social complexity in three species of Hymenoptera. We utilized a species triad to compare two species that were either similar (*Polistes* or *Bombus*) or different (*Polistes* and *Apis*), while controlling for phylogenetic distance. We found that *Polistes* and *Bombus*, two less socially complex species that evolved eusociality independently, shared greater patterns of adaptive molecular evolution relative to *Polistes* and *Apis*. This includes sharing more genes under positive selection, and greater rates of positive selection acting on queen-biased genes. Our study also highlighted the importance of adaptive changes in gene regulation during the earliest stages of social evolution; genes involved in regulating transcription were highly enriched among adaptively evolving genes in *Polistes*.

## Methods

### Genome Alignment, Variant Calling, and Filtration

Methods for genome alignment, SNP detection, and filtering are described in detail in the supplementary material. In brief, paired-end Illumina genome sequencing was performed on ten *P*. *dominula* and two *P*. *gallicus* female worker samples. Reads were aligned to the unmasked *P*. *dominula* reference genome (PdomGDB r1.2)^33^ using BWA-MEM^47^. Duplicates were marked using Picard (http://broadinstitute.github.io/picard/) and indel realignment was performed using the Genome Analysis Toolkit (GATK)^48,49^. Variants were detected with GATK’s HaplotypeCaller using all species-specific alignments in unison. Variants were subsequently filtered of poor quality SNPs based on GATK’s hard filter recommendations, upper and lower depth limits, missing data, and regions of high sequence homology. In addition, genes were filtered on a percent threshold for low to no coverage across coding regions. Genes that had poor coverage (based on lower depth limit) for >0.1 (10%) of the coding sequence were removed from further analysis.

### Variant Annotation and Filtration

Variants that passed all of the filtering criteria were annotated for predicted effects on genes using SnpEff ^50^. Genes were removed from the analysis if they contained warnings for an incomplete transcript, multiple stop codons, and no start codon. Additionally, genes were removed if they possessed annotations for lost stop codons, gain of stop codon, loss of start codon, or non-synonymous start variants. Tri-allelic variants were also discarded to limit the chances of retaining SNPs with potential sequencing error and to simplify downstream analysis for the large dataset^51^.

### Quantifying Selection

A Bayesian implementation of the McDonald-Kreitman test^23^ was used to estimate the prevalence of selection acting on genes in *Polistes*. Synonymous and non-synonymous substitutions were determined using the predicted gene annotations from SnpEff. Divergence data was based on fixed mutations between *P*. *dominula* and *P*. *gallicus* gene sequences, while polymorphisms were based on variable mutation sites in both species. Sites were removed if the comparison resulted in a triallelic variant and in cases where both species were fixed for the same allele. The Bayesian implementation of the McDonald-Kreitman test, SnPIRE, makes use of genome wide information and doesn’t require a priori knowledge of species divergence parameters. The selection coefficient, gamma (γ), is calculated for each gene, and represents the average selection coefficient on non-synonymous mutations in a gene scaled by the effective population size (γ = 2N_e_s) (Eliertson *et al*. 2012). Typically, γ > 1 indicates positive selection^23^. Selection coefficients for *Apis* and *Bombus* were collected from previously published datasets^16,24^.

### Gene ontology

Gene ontology (GO) was performed using DAVID 6.8 (2013–2017)^52^ with default annotation databases including KEGG pathways and Interpro protein domains. We performed the analysis using *Drosophila melanogaster* fly base gene IDs. *Polistes* sequences orthologous to *Drosophila* were identified using reciprocal blastp matches with an E-value threshold of 1e^−10^. *Drosophila* orthologs were found for 6534 genes (55%) of the 11815 annotated genes in *Polistes* (PdomGDB r1.2)^33^. Reciprocal blastp matches were also found for *Apis* (n = 6986) and *Bombus* (n = 12393) using the same method. Gene ontology functional annotation clusters with an enrichment score > = 1.3, and gene ontology terms with p < 0.05 after FDR correction were determined to be of significance.

### Differential Gene Expression

Difference in gene expression between *Polistes* queens and workers was determined from Illumina paired-end RNA-Seq libraries of six *P*. *dominula* queen (SRX1124061, SRX1124060, SRX1124059, SRX1124057, SRX1124056, SRX1124054) and five *P*. *dominula* worker (SRX1124053, SRX1124052, SRX1124051, SRX1124049, SRX1122234) whole heads^33^. Sequences were downloaded from GenBank and aligned to the unmasked *P*. *dominula* reference genome (PdomGDB r1.2)^33^ using the default parameters of STAR’s 2-pass method^53^. The STAR 2-pass method performs two alignments in which the splice junctions detected in the first alignment are used to guide the second alignment. Using the resulting BAM files, transcriptomes were assembled using Cufflinks^54^. Transcriptomes were produced with bias detection and correction algorithms using the *P*. *dominula* reference sequence (PdomGDB r1.2)^33^, and were quantified using the annotated reference genome. Assembled transcriptomes were merged using Cuffmerge to concatenate overlapping regions that agree in splice and orientation position. Cuffdiff was then used to find significant changes in gene level expression between worker and queen castes. Loci were removed from the analysis if they could not be identified after the merge step due to: the direct overlap of genes located on opposite strands, genes on the same strand that were merged due to shared transcripts, or clustering of three of more genes. Remaining genes were determined to be significantly differentially expressed if they passed the following thresholds: FDR < 0.05, and FPKM > 1. Differential expression data for *Apis mellifera* (i.e. the honey bee protein atlas which lists proteins with consistent patterns of differential expression across 26 tissues of queens and workers) and *Bombus impatiens* (i.e. microarray study that examined brain gene expression of queens, workers, foundresses, and gynes) were collected from previously published datasets^41,55^. We followed the same procedure used to previously identify caste biased genes^16,24^. Our study makes use of the best transcriptomic data sets currently available for *Apis*, *Bombus*, and *Polistes*, but clearly, the honey bee data set is much larger in scope relative to *Bombus* and *Polistes*. Studies of positive selection on caste biased genes will surely improve as richer transcriptomic data sets become available for *Polistes* and *Bombus*.

### Ortholog analysis between *Polistes*, *Bombus*, and *Apis*

We conducted a cross-species orthology analysis involving *Polistes*^33^, *Bombus*^56^, and *Apis*^57^. Orthologs between species pairs were identified using reciprocal blastp matches with an E-value threshold of 1e^−10^. Multiple best blastp matches of the same sequence segment were considered. Reciprocal matches with corresponding gamma values for *Polistes* and *Apis*^16^, *Polistes* and *Bombus*^24^, and *Bombus* and *Apis*, revealed 7338, 6496, and 8204 ortholog matches respectively. A three-way reciprocal blastp between all three species produced 5943 gene orthologs.

### Data availability

All sequence data used in this paper are available from NCBI’s Sequence Read Archive (SRA) under BioProject PRJNA477544.

## Electronic supplementary material

SUPPLEMENTARY INFO

Supplementary Dataset 1
