# Supplementary material for: Insects with similar social complexity show convergent patterns of adaptive molecular evolution
Source: Sci Rep. 2018 Jul 10;8:10388. doi: 10.1038/s41598-018-28489-5 (PMC6039441; doi:10.1038/s41598-018-28489-5)
Supplement: Supplementary file 1 — SUPPLEMENTARY INFO [file 41598_2018_28489_MOESM1_ESM.docx]

**Title:** Insects with similar social complexity show convergent patterns of adaptive molecular evolution

**Authors:**

Kathleen A. Dogantzis^1^, Brock A. Harpur^1,2^, André Rodrigues^3^, Laura Beani^4^, Amy L. Toth^5^, Amro Zayed^1*^

1. Department of Biology, York University. 4700 Keele St., Toronto, Ontario, Canada.
2. Donnelly Centre, University of Toronto. Toronto, Canada.
3. Departamento de Biologia, Faculdade de Filosofia Ciências e Letras de Ribeirão Preto, Universidade de São Paulo, São Paulo, Brazil
4. Dipartimento di Biologia, Università di Firenze, Italia
5. Department of Ecology, Evolution & Organismal Biology and Entomology, Iowa State University. Ames, United States.

* Corresponding Author: [zayed@yorku.ca](mailto:zayed@yorku.ca)

## Methods

### Sample Preparation and Whole Genome Sequencing

Samples of *Polistes dominula* and its sister species *Polistes gallicus* were collected within their native ranges in Tuscany, Italy in August 2014. Samples were subsequently sequenced for DNA using extractions from the whole thorax. Whole genome sequencing was performed on ten *P. dominula* and two *P. gallicus* samples on the Illumina HiSeq 2500 system using two lanes per sample. Library preparation and sequencing was performed at The Centre for Applied Genomics (Toronto, On). The use of 24 chromosomes for conducting the McDonald-Kreitman test provides an appropriate amount of statistical power for calculating the selection coefficient (𝛄) ^1^. Similar samples sizes have been used in previous studies ^2,3^.

### Whole genome alignment

Illumina paired-end reads were trimmed of adapters and poor quality bases using the default settings of Trimmomatic^4^. Reads (Raw FASTQ) were then aligned to the unmasked *P. dominula* reference sequence (PdomGDB r1.2)^5^ using the default parameters of the Burrows-Wheeler aligner MEM algorithm^6^. Alignments were sorted and output as BAM files using Samtools^7^. The sorted BAM files were marked for duplicates with validation stringency set to silent, and read groups were replaced using Picard (http://broadinstitute.github.io/picard/). Subsequent files were indexed to BAM files using Samtools. The resulting BAM files were realigned around indels using default parameters in GATK (3.5-0-g36282e)^8^. Quality and coverage for each of the BAM files was assessed using QualiMap^9^ (Supplementary Table S1).

### Whole Genome Variant Calling and Filtration

Variants were detected using GATK’s HaplotypeCaller using all species-specific BAM files in unison. Variants identified with GATK were initially filtered for a Minimum Base Quality (MBQ) (MBQ > 20), followed by filtering based on the variant call annotations produced in the subsequent VCF file. Filtering was performed using GATKs hard filter recommendations^10,11^. SNPs (single nucleotide polymorphisms) were discarded (Fail SNPs) if they had a poor Mapping Quality (MQ < 40) or inconsistent base qualities between the reference and alternative alleles (MQRankSum < -12.5), and if there was strand (FS > 60) or position bias (ReadPosRankSum < -8.0) for alternative allele calls. SNPs were also discarded if there was poor variant confidence (QD < 5) and if they had an unusually high or low depth of coverage (DP < 100 & > 350 for *P. dominula*, and DP < 20 & > 83 for *P. gallicus*). Upper limit thresholds for depth of coverage were determined by calculating the 1.5*IQR (interquartile range) for the total depth of coverage for each variant. The lower threshold was determined by allowing an average depth of coverage of ten reads per base pair per individual. SNPs positioned within and around five base pairs of unfiltered indels were also discarded. From the variants remaining, SNPs were filtered based on minimum frequency threshold for missing data. Since there was a small sample size, any variant that had missing data was discarded from the analysis. Additionally, regions with highly repetitive sequences or recently duplicated genes were filtered from the analysis. Highly homologous regions were identified by dividing the reference genome of *P. dominula* into 150-bp fragments and using Blastn to match those segments back onto the reference genome. SNPs that were present within segments which resulted in a Blastn match to two or more regions with a corresponding E-value of 10e^-40^ or higher were removed from the analysis. Lastly the discarded SNPs (Fail SNPs) from *P. gallicus* were removed from *P. dominula* and vice versa to avoid skewing fixed and polymorphic variant ratios by retaining potentially poor quality SNPs. All remaining SNPs (Pass SNPs) were used in subsequent analyses (Supplementary Table S2, S3).

***Overview***

Post filtering, there were 1,941,335 polymorphic SNPs in the *P. dominula* dataset (Table 2) and 2,903,697 polymorphic SNPs and fixed divergences in the *P. gallicus* dataset (Table 3). We removed 506 genes due to incomplete transcripts, multiple stop codons, or no start codon, and 1641 genes due to a loss of stop codon, gain of stop codon, loss of start codon, non-synonymous start variants, non-synonymous stop variants, or poor gene coverage. The 9668 remaining genes were used to conduct the population genomic analyses.

**Supplementary Table S1** – Average depth of coverage of each aligned BAM file.

| Sample ID | Number of reads | Average Depth of Coverage |
| --- | --- | --- |
| *Polistes dominula* 1 | 42,695,484 | 23.1208X |
| *Polistes dominula* 2 | 56,445,842 | 31.1812X |
| *Polistes dominula* 3 | 51,598,409 | 28.4135X |
| *Polistes dominula* 4 | 46,221,292 | 25.5051X |
| *Polistes dominula* 5 | 46,494,397 | 25.5627X |
| *Polistes dominula* 6 | 38,802,766 | 21.1769X |
| *Polistes dominula* 7 | 51,051,071 | 28.2273X |
| *Polistes dominula* 8 | 51,051,365 | 28.4032X |
| *Polistes dominula* 9 | 47,877,121 | 26.4893X |
| *Polistes dominula* 10 | 51,078,088 | 27.0433X |
| *Polistes gallicus* 4 | 49,163,384 | 24.2483X |
| *Polistes gallicus* 8 | 40,537,862 | 20.1191X |

**Supplementary Table S2** – Number of sites removed after applying each filter for *Polistes dominula* (some SNPs have multiple filters).

| Filters |  |  | Number of SNPs |
| --- | --- | --- | --- |
|  | **GATK Command** | **Raw Variants:** | SNPS: **3,105,907** |
| INDELS & Mask Extension 5 | -mask –maskExtension 5 |  | - 263,291 |
| Mapping Quality | MQ < 40 |  | - 57,946 |
| Fisher Strand | FS > 60 |  | - 25,529 |
| Quality by Depth | QD < 5 |  | - 171,927 |
| Mapping Quality Rank Sum | MQRankSum < -12.5 |  | - 13,860 |
| Read Position Rank Sum | ReadPosRankSum < -8.0 |  | - 2,636 |
| Min Depth of Coverage | DP < 100 |  | - 257,005 |
| Max Depth of Coverage | DP > 350 |  | - 151,647 |
| Missing Genotypes |  |  | - 100,884 |
| Repetitive regions |  |  | - 351,535 |
| Discarded overlapping SNPs |  |  | - 47,172 |
|  |  | **Total Removed:** | **1,164,572 or 37%** |
|  |  | **Total Remaining:** | **1,941,335** |

**Supplementary Table S3** – Number of sites removed after applying each filter for *Polistes gallicus* (some SNPs have multiple filters).

| Filters |  |  | Number of SNPs |
| --- | --- | --- | --- |
|  | **GATK Command** | **Raw Variants:** | SNPS: **3,998,783** |
| INDELS & Mask Extension 5 | -mask –maskExtension 5 |  | - 293,116 |
| Mapping Quality | MQ < 40 |  | - 181,782 |
| Fisher Strand | FS > 60 |  | - 2,082 |
| Quality by Depth | QD < 5 |  | - 36,766 |
| Mapping Quality Rank Sum | MQRankSum < -12.5 |  | - 67 |
| Read Position Rank Sum | ReadPosRankSum < -8.0 |  | - 44 |
| Min Depth of Coverage | DP < 20 |  | - 457,529 |
| Max Depth of Coverage | DP > 83 |  | - 61,027 |
| Missing Genotypes |  |  | - 37,768 |
| Repetitive regions |  |  | - 211,196 |
| Discarded overlapping SNPs |  |  | - 38,033 |
|  |  | **Total Removed:** | **1,095,086 or 27%** |
|  |  | **Total Remaining:** | **2,903,697** |

Supplementary Figure S1: Distribution of the selection coefficient (γ) for 9668 protein coding genes in the *Polistes* genome. Segments highlighted in orange indicate loci experiencing adaptive evolution (γ > 1).

1 Andolfatto, P. Controlling type-I error of the McDonald–Kreitman test in genomewide scans for selection on noncoding DNA. *Genetics* **180**, 1767-1771 (2008).

2 Harpur, B. a. *et al.* Population genomics of the honey bee reveals strong signatures of positive selection on worker traits. *Proceedings of the National Academy of Sciences of the United States of America* **111**, 2614-2619, doi:10.1073/pnas.1315506111 (2014).

3 Harpur, B. A. *et al.* Queens and workers contribute differently to adaptive evolution in bumble bees and honey bees. *Genome Biology and Evolution* **evx182** (2017).

4 Bolger, A. M., Lohse, M. & Usadel, B. Trimmomatic: a flexible trimmer for Illumina sequence data. *Bioinformatics*, btu170 (2014).

5 Standage, D. S. *et al.* Genome, transcriptome and methylome sequencing of a primitively eusocial wasp reveal a greatly reduced DNA methylation system in a social insect. *Molecular Ecology* **25**, 1769-1784, doi:10.1111/mec.13578 (2016).

6 Li, H. Aligning sequence reads, clone sequences and assembly contigs with BWA-MEM. *arXiv preprint arXiv:1303.3997* (2013).

7 Li, H. A statistical framework for SNP calling, mutation discovery, association mapping and population genetical parameter estimation from sequencing data. *Bioinformatics* **27**, 2987-2993 (2011).

8 McKenna, A. *et al.* The Genome Analysis Toolkit: a MapReduce framework for analyzing next-generation DNA sequencing data. *Genome research* **20**, 1297-1303 (2010).

9 Okonechnikov, K., Conesa, A. & García-Alcalde, F. Qualimap 2: advanced multi-sample quality control for high-throughput sequencing data. *Bioinformatics* **32**, 292-294 (2015).

10 DePristo, M. A. *et al.* A framework for variation discovery and genotyping using next-generation DNA sequencing data. *Nature genetics* **43**, 491-498, doi:10.1038/ng.806 (2011).

11 Auwera, G. A. *et al.* From FastQ data to high‐confidence variant calls: the genome analysis toolkit best practices pipeline. *Current protocols in bioinformatics*, 11.10. 11-11.10. 33 (2013).
